# High Influenza Vaccine Effectiveness and Absence of Increased Influenza-like-Illness Epidemic Activity in the 2021–2022 Influenza Season in Catalonia (Spain) Based on Surveillance Data Collected by Sentinel Pharmacies

**DOI:** 10.3390/vaccines10122140

**Published:** 2022-12-14

**Authors:** Pedro Plans Rubió, Anna M. Jambrina, Pilar Rius, Gloria Carmona, Manel Rabanal, Montse Gironès

**Affiliations:** 1 Public Health Agency of Catalonia, Health Department of Catalonia, 08005 Barcelona, Spain; 2 CIBER of Epidemiology and Public Health (CIBERESP), 28028 Madrid, Spain; 3 General Directorate for Healthcare Planning and Regulation, Department of Health of Catalonia, 08028 Barcelona, Spain; 4 Faculty of Pharmacy and Food Science, University of Barcelona, 08028 Barcelona, Spain; 5 Council of the Pharmacists’ Association of Catalonia, 08009 Barcelona, Spain

**Keywords:** influenza vaccination, influenza surveillance, influenza-like illness, influenza epidemic, community pharmacies, sentinel pharmacies

## Abstract

Influenza surveillance and influenza vaccination are the key activities for preventing and controlling influenza epidemics. The study assessed the influenza surveillance and influenza vaccination data obtained from sentinel pharmacies of Catalonia, Spain, in the 2021–2022 influenza season. The sentinel pharmacies were selected from all community pharmacies to report all influenza-like illness (ILI) cases detected during the 2021–2022 influenza season and collect influenza surveillance and influenza vaccination data. The ILI cases were identified based on European Centre for Disease Control (ECDC) criteria. The moving epidemic method (MEM) was used to assess the ILI epidemic activity. The screening method was used to assess influenza vaccination effectiveness in patients aged 65-or-more years old. The sentinel pharmacies reported 212 ILI cases with a negative COVID-19 test and a total number of 412 ILI cases. An absence of increased ILI epidemic activity was observed in the 2021–2022 influenza season based on two criteria: (1) Number of ILI cases reported per week in the 2021–2022 influenza season significantly lower than the MEM-based epidemic threshold. (2) Mean number of ILI cases reported per week in the 2021–2022 influenza season significantly lower than during the ILI/influenza epidemic periods detected from 2017 to 2020 using the same methodology. Influenza vaccination was effective in preventing ILI among patients aged 65-or-more-years old. The absence of the influenza epidemic during the 2021–2022 influenza season could be explained by influenza vaccination and COVID-19 prevention measures (wearing face masks, social distancing). The sentinel pharmacies provided influenza surveillance data not provided by traditional influenza surveillance systems.

## 1. Introduction

Influenza is responsible for a seasonal epidemic every winter in the Northern and Southern hemispheres, with a great impact on the health system due to morbidity, mortality and utilization of health resources. Community pharmacies are involved in the prevention and treatment of influenza in Spain by developing the following activities: the dispensation of anti-influenza medications and influenza vaccines, review of medications and drug interactions, support to primary health care and specialized services, and health education to promote influenza vaccination [1,2].

In the future, the activities developed by community pharmacies could also include the administration of influenza vaccines and influenza surveillance [1,2,3]. Several studies have found higher percentages of influenza vaccination coverage in countries and regions where influenza vaccines are administered in community pharmacies [4,5,6]. Traditional influenza surveillance is based on the collection and analysis of data obtained from hospitals, primary health care centers and sentinel physicians [7].

In 2017, a new influenza surveillance system based on the sentinel pharmacies was developed in Catalonia, a region of Spain with 7.5 million inhabitants [8,9,10]. The objective of the new surveillance system was to obtain information about the influenza epidemic and influenza surveillance data not provided by traditional surveillance systems. The activities developed by sentinel pharmacies are important to achieve high percentages of influenza vaccination coverage for several reasons. First, the influenza surveillance data collected by sentinel pharmacies can highlight the importance of influenza vaccination among individuals aged 65 years or more and those with chronic conditions. Second, pharmacists can develop health education activities to promote influenza vaccination in the population and among individuals aged 65 years or more and those with chronic conditions. Third, in the future, community pharmacies can administer influenza vaccines in Spain [1,2].

The objectives of this study were: (1) to analyze the influenza surveillance data obtained from sentinel pharmacies in the 2021−2022 influenza season and (2) to assess the effectiveness of influenza vaccination among patients aged 65 years or more in the 2021–2022 influenza season.

## 2. Materials and Methods

### 2.1. Influenza-Like Illness Surveillance Based on Sentinel Pharmacies

Influenza surveillance information was collected from a representative sample of community pharmacies in Catalonia. Sixty sentinel pharmacies were selected from the list of community pharmacies of Catalonia in two phases [10]. In the first phase, a cluster and population analysis were carried out to determine the number of community pharmacies required to ensure the representativeness of the population to be monitored. In the second phase, 60 community pharmacies were selected by taking into account the results of the cluster and population analysis and the percentage of the total population of Catalonia in each province.

The sentinel pharmacies participating in the study should detect and report all influenza-like illness (ILI) patients attended during the influenza season (from week 40 of 2021 to week 20 of 2022) and collect socio-demographic and influenza-related health information from ILI patients. The ILI cases were identified based on the European Centre for Disease Prevention and Control (ECDC) case definition [11]: (1) sudden onset of symptoms; (2) fever; (3) at least one of three systemic symptoms: malaise, headache, muscle pain (myalgia); and (4) at least one of three respiratory symptoms: cough, sore throat, shortness of breath. An anonymous questionnaire, accessible on-line, was used to collect the following information from ILI cases: age and sex, ILI symptoms, medications dispensed by pharmacies, previous medical visits, medications prescribed by physicians, negative result for a COVID-19 detection test, derivation from the pharmacy to a primary heath care center and influenza vaccination. The individuals were considered vaccinated during the 2021–2022 influenza season when they had received the influenza vaccine 14 days or more before symptom onset.

The ILI cases reported by sentinel pharmacies were classified into two categories: (1) ILI cases with a negative result for a COVID-19 detection test and (2) ILI cases that had not undertaken a COVID-19 test. The COVID-19 tests included the antigen test and PCR tests. The negativity to COVID-19 was confirmed by reviewing the official document or the test result for self-administered tests and tests undertaken in the pharmacy. The sentinel pharmacies did not report patients with COVID-19, a positive COVID-19 test, ILI patients who were contacts of patients with COVID-19 or individuals with a positive COVID-19 test.

The influenza surveillance data obtained from sentinel pharmacies was assessed for all ILI cases reported and for ILI cases with a negative result for a COVID-19 detection test. The analysis carried out for ILI cases with a negative COVID-19 test avoids the potential overestimation of ILI cases due to SARS-CoV-2 infections among ILI cases not undertaking a COVID-19 detection test.

Verbal informed consent was obtained from all influenza-like patients or their parents to participate in the study and collect sociodemographic and influenza-related health information using an anonymous questionnaire. The study was approved by the Health Department of Catalonia, the Public Health Agency of Catalonia and the Council of the Pharmacists’ Association of Catalonia.

### 2.2. Analysis of the Influenza-Like-Illness Activity

Weekly numbers of ILI cases reported by sentinel pharmacies were used to analyze the influenza epidemic in the 2021–2022 influenza season. The absence of influenza epidemic activity during the 2021–2022 influenza season could be detected based on two criteria: (1) Number of ILI cases reported per week during the 2021–2022 influenza season lower than the epidemic threshold. (2) Mean number of ILI cases reported per week in 2021–2022 significantly lower than the mean number of cases reported per week during the ILI/influenza epidemic periods detected in the 2017–2018, 2018–2019 and 2019–2020 influenza seasons using the same methodology.

The moving epidemic method (MEM) [8,12] was used to obtain the epidemic threshold, in terms of ILI cases reported per week, for detecting epidemic activity during the 2021–2022 influenza season. The MEM-based thresholds by basal, moderate and intense epidemic activity were determined using the influenza surveillance data collected during previous influenza seasons with epidemic activity (2017–2020). ILI epidemic activity occurs when the number of cases reported per week is higher than the epidemic threshold. The influenza epidemic begins when the number of reported ILI cases is higher than the epidemic threshold and finishes when it is again lower than the epidemic threshold.

The method used in this study to detect increased ILI activity and ILI epidemics was validated by assessing the data obtained from sentinel pharmacies in the 2017–2018, 2018–2019 and 2019–2020 influenza seasons [8].

### 2.3. Influenza Vaccination Effectiveness

In Spain, influenza vaccination is recommended among individuals aged 65 years or more [13]. The effectiveness of the influenza vaccination (VE) in preventing ILI among individuals aged ≥65 years in the 2021–2022 season was calculated using the screening method [14]. The influenza vaccination effectiveness (VE) was determined using the formula: VE = (PV − PCV)/(PV (1 − PCV)). In this formula, PV is the influenza vaccination coverage in individuals aged ≥65 years in Catalonia in the 2021–2022 influenza season and PCV is the proportion of ILI cases aged ≥65 years vaccinated against influenza.

### 2.4. Statistical Analysis

The percentages and their 95% confidence intervals were determined for qualitative study variables in different groups. The Chi-square test (Fisher’s exact test when necessary) and the odds ratios were used to compare percentages in different groups, considering a *p* < 0.05 as statistically significant. The t test was used to compare the mean number of ILI cases reported per week in the 2021–2022 influenza season with the mean number of ILI cases reported in the 2017–2018, 2018–2019 and 2019–2020 influenza seasons. A *p* < 0.05 was considered as statistically significant. The statistical analysis was carried out using the IBM-SPSS program (Version 18, IBM-SPSS, Chicago, IL, USA).

## 3. Results

### 3.1. Influenza-Like-Illness Cases Reported by Sentinel Pharmacies

Fifty-three (88.3% participation rate) sentinel pharmacies reported 212 influenza-like illness (ILI) cases with a negative result for a COVID-19 detection test and a total number of 402 ILI cases. The distribution of sentinel pharmacies by province in 2022–22 was: 31 (58.4%) from Barcelona, 8 (13.5%) from Tarragona, 9 (17%) from Girona and 5 (9.6%) from Lleida. The percentage of pharmacies in each province was not different from the percentage of the total population of Catalonia in each province [10,15].

For the ILI cases with a negative COVID-19 test, 103 (48.6%) were men and 109 (51.4%) were women (Table 1). The mean age of the ILI cases with a negative COVID-19 test was 38.4 years, 34.2 years in men and 42.2 years in women (*p* < 0.005). The percentage of ILI cases with a negative COVID-19 test aged 15–64 years was significantly higher than that for cases aged <15 years or ≥65 years (78.8% vs. 21.2%, *p* < 0.001). The OR for cases aged 15–64 years against cases aged <15 years or ≥65 years was 13.8 (95% CI: 8.6–21.9).

For all ILI cases reported by sentinel pharmacies, 200 (49.8%) were men and 202 (50.2%) were women (Table 1). The mean age for all ILI cases was 37.8 years, 35.5 years in men and 40.1 years in women (*p* < 0.05). The percentage of ILI cases aged 15–64 years was significantly higher than that for cases aged <15 years or ≥65 years (78.4% vs. 21.6%, *p* < 0.001). The OR for cases aged 15–64 years against cases aged <15 years or ≥65 years was 13.1 (95% CI: 9.37–18.33). The distribution of ILI cases with a negative COVID-19 test by age was similar to the distribution for all ILI cases.

### 3.2. Influenza-Like-Illness Epidemic Activity

The influenza epidemic threshold for the 2021–2022 influenza season, determined using the MEM method and influenza surveillance data in the previous influenza seasons with an influenza epidemic (2017–2020), was 29.79 ILI cases per week. The epidemic thresholds for medium, high and very high epidemic activity were 51.63, 133.8 and 203.7 ILI cases per week, respectively.

**Figure 1:**
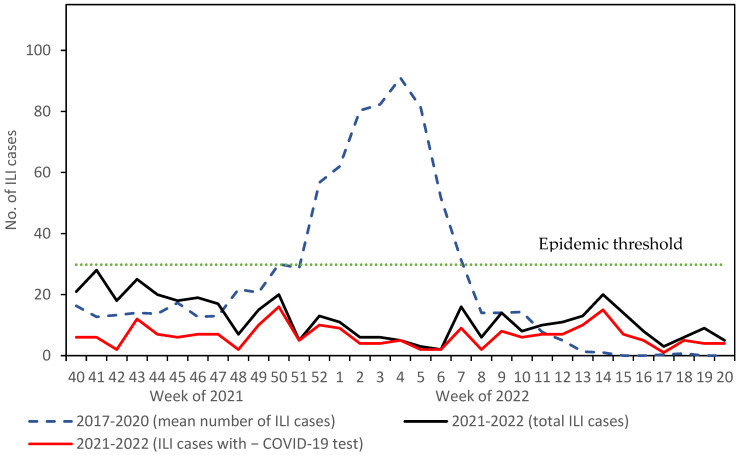
Number of influenza-like illness (ILI) cases reported per week in the 2021–2022 influenza season and mean number of ILI cases reported per week in 2017–2020. The epidemic threshold for the 2021–2022 influenza season was determined using the moving epidemic method (MEM) and influenza surveillance data obtained from sentinel pharmacies for the 2017–2018, 2018–2019 and 2019–2020 influenza seasons [8].

The study found a low ILI activity during the 2021–2022 influenza season based on surveillance data obtained from sentinel pharmacies (Figure 1). The study found that the influenza-like illness/influenza epidemic did not occur in the 2021–2022 influenza season based on two criteria. First, the number of cases reported per week was lower than the epidemic threshold of 29.79 for ILI cases with a negative COVID-19 test and for all ILI cases (Figure 1). Second, the mean number of ILI cases with a negative COVID-19 test reported per week in the 2021–2022 influenza season (6.4; SD: 3.6) was significantly lower than that in the epidemic waves of 2017–2018 (72.8; SD: 38.7; *p* < 0.001), 2018–2019 (71.3; SD: 39.7; *p* < 0.001) and 2019–2020 (62.8; SD: 29.1; *p* < 0.001). The mean number of all ILI cases reported per week (12.2; SD: 6.9) was also significantly lower than in the three epidemic waves observed in prior influenza seasons (2017–2020).

At the end of the influenza season, the weekly numbers of ILI cases reported by sentinel pharmacies were higher than during 2017–2020, but they were not higher than the epidemic threshold (Figure 1).

### 3.3. Influenza Vaccination Effectiveness

Next, 6.6% of the ILI patients with a negative COVID-19 test and 8% of all the ILI patients reported by sentinel pharmacies had been vaccinated against influenza in the 2021–2022 influenza campaign (Table 2). Among individuals aged 65 years or more, the influenza vaccination coverage was 30% in ILL cases with a negative COVID-19 test and 30.5% in all ILI cases. The influenza vaccination coverage was significantly higher in ILI cases with a negative COVID-19 test aged 65 or more years than in individuals aged <65 years (30% vs. 5.7%, *p* < 0.005), with an OR of 7.05 (95% CI: 2.36–21.33). The influenza vaccination coverage was also significantly higher in all ILI cases aged 65 years or more than in those aged <65 years (30.5% vs. 3.7%, *p* < 0.001), with an OR of 11.95 (95% CI: 4.95–28.9).

Six (29.4%) of the ILI patients with a negative COVID-19 test vaccinated against influenza had received the influenza vaccine for the recommendation addressed to people aged 65 years or more; two (11.8%) for the recommendation addressed to people with high risk of influenza complications; three (17.6%) for the recommendation addressed to health professionals; one (4.2%) for the recommendation addressed to caregivers of people with high risk of influenza complications; and five (29.4%) had been vaccinated for personal reasons. Among all the ILI patients vaccinated against influenza, 11 (45.8%) patients had received the influenza vaccine for the recommendation addressed to people of 65 or more years; 4 (16.7%) for the recommendation addressed to people with a high risk of influenza complications; 3 (12.5%) for the recommendation addressed to health professionals; 1 (4.2%) for the recommendation addressed to caregivers of people with high risk of influenza complications; and 5 (20.8%) had been vaccinated for personal reasons.

The effectiveness of the influenza vaccination in individuals aged ≥65 years in preventing ILI with a negative COVID-19 test was 76% and the effectiveness in preventing ILI with or without a negative COVID-19 test was 75.4% (Table 2). The effectiveness of influenza vaccination was determined taking into account the influenza vaccination coverage in individuals aged ≥65 years (64.1%) in Catalonia in the 2021–2022 influenza season [13].

The effectiveness of the influenza vaccination in individuals aged ≥65 years obtained in this study for the 2020–2021 influenza season was higher than the effectiveness observed in the 2018–2019 (51.4%), 2019–2020 (67.1%) and 2020–2021 (53.7%) influenza seasons [8,9], although the differences were not statistically significant.

### 3.4. Medications Dispensed and Prescribed to Influenza-Like Illness Patients

Sentinel pharmacies dispensed medications to 92.5% of the ILI patients with a negative COVID-19 test, 89.3% in men and 96% in women (Table 3). The percentage of dispensation to ILI cases ranged from 87.5% in individuals aged 5–14 years to 95% in those aged 65 years or more. Nevertheless, the differences among age groups were not statistically significant.

Four medications were dispensed to 20% of more of the ILI patients with a negative COVID-19 test: paracetamol (76.4%), cough medications (31.6%), ibuprofen (21.7%) and antihistamines (21.2%) (Table 4). Antibiotics were dispensed to 6.6% and oseltamivir to 0.5% of the ILI patients with a negative COVID-19 test.

The percentage of the ILI cases with a negative COVID-19 test that had received a previous medical visit was 44.3%, 41.7% in men and 46.8% in women (Supplementary Materials Table S1). The physicians had prescribed medications to 38.2% of the ILI patients with a negative COVID-19 test, 37.9% in women and 38.5% in men (Table 3). Five medications were prescribed to 5% of more ILI patients: paracetamol (30.2%), ibuprofen (10.4%) cough medication (9%), antihistamines (7.5%) and antibiotics (6.6%) (Table 4). Oseltamivir was prescribed to 0.5% of the ILI patients with a negative COVID-19 test (Table 4).

The sentinel pharmacies dispensed medications to 93.8% of all ILI patients (with or without a negative COVID-19 test), 94.6% in men and 93% in women (Supplementary Materials Table S2). Four medications were dispensed to 20% of more of ILI patients: paracetamol (76.6%), cough medications (27.4%), antihistamines (22.1%) and ibuprofen (20%) (Supplementary Materials Table S3). Antibiotics were dispensed to 4.7% and oseltamivir to 0.2% of the ILI patients.

The physicians had prescribed medications to 30.2% of ILI patients with or without a negative COVID-19 test, 29.5% in women and 31.2% in men (Supplementary Materials Table S2). Four medications were prescribed to 5% of more of the ILI patients: paracetamol (23.9%), ibuprofen (9.2%) cough medication (7.7%) and antihistamines (5.0%). Antibiotics were prescribed to 3.4% and oseltamivir to 0.2% of the ILI patients (Supplementary Materials Table S3).

The percentages of dispensation of medications, paracetamol, oseltamivir and antibiotics in ILI patients with a negative COVID-19 test were similar to those for all ILI patients.

## 4. Discussion

The study found that influenza vaccination effectiveness in preventing ILI was high among individuals aged 65 or more years and ILI epidemic activity did not increase significantly during the 2021–2022 influenza season.

The lack of increased ILI activity during the 2021–2022 influenza season could be explained by several factors. First, influenza vaccination among individuals aged 65 or over was high in Catalonia (64.1%) and Spain (69.4%) during the 2021–2022 influenza season [13]. Second, the study found that influenza vaccination was 75–76% effective in preventing ILI among patients aged 65 or more years. Third, COVID-prevention measures implemented during 2022 must have been effective in preventing the transmission of influenza and other respiratory viruses [16,17]. In Spain, wearing face masks and social distancing were obligatory during 2022 in health centers, pharmacies, nursing homes, public transport, indoor public places, schools, entertainment and places of workshop. Since 20 April 2022, wearing face masks was obligatory only in health centers, pharmacies, nursing homes and public transport. COVID-19 prevention measures applied during 2022 could have contributed also to increase influenza vaccine effectiveness.

The influenza vaccination effectiveness found in this study was higher than that found among individuals aged ≥6 months (34%, 95% CI: 19–46%) in the United States of America [18,19]. Nevertheless, it is difficult to compare the effectiveness found in this study with that found in the USA due to methodological, epidemiological and influenza vaccination coverage differences. In the study carried out by the CDC, the effectiveness was measured in terms of preventing medically attended laboratory-confirmed influenza A, the sample studied included individuals aged 6 months or more attended in health centers and the vaccination effectiveness was determined using multivariable logistic regression models adjusted for site, age, month of onset, self-rated general health status and race/ethnicity [18]. During the 2021–2022 season, the influenza vaccination coverage in individuals aged 65 years or more was 75% in the United States [19].

In this study, the effectiveness of the influenza vaccine was measured in terms of the prevention of ILI. The main advantages of using ILI for assessing influenza vaccination effectiveness are that it is easy to apply the ILI case definition in the pharmacy setting and that the laboratory-confirmation of influenza requires biological sampling, time and resources. The main disadvantage is that the effectiveness in preventing ILI must be lower than for laboratory-confirmed influenza because other respiratory viruses are associated with ILI [20]. Nevertheless, during the influenza epidemic wave, influenza is more frequent than other respiratory pathogens among ILI patients [21].

In this study, the absence or lack of increased ILI epidemic activity during the 2021–2022 influenza season was detected based on two criteria: (1) The number of ILI cases reported per week was lower than the MEM-based epidemic threshold during the season. (2) The mean number of ILI cases reported per week by sentinel pharmacies was significantly lower than the mean number of cases reported per week during the ILI/influenza epidemic waves detected during 2017–2020 using the same methodology. The low number of ILI cases reported by sentinel pharmacies in Catalonia in the 2021–2022 influenza season was consistent with the low influenza activity detected by traditional influenza surveillance systems. In Catalonia, in the 2021–2022 influenza season, the influenza incidence was lower than 50 per 100.000 during the season except from week 11 to 19 of 2022 (peak of 121 per 100.000) and only 44 cases of influenza A were detected among 764 patients hospitalized in sentinel hospitals due to severe acute respiratory infection [22,23]. The influenza wave detected by other influenza surveillance systems at the end of the influenza season could not be detected by the influenza surveillance system based on sentinel pharmacies for two reasons: (1) The epidemic threshold for detecting influenza/ILI epidemics was determined using data from prior influenza seasons (2017–2020) with influenza/ILI epidemics. (2) The number and distribution of sentinel pharmacies in the territory could not be sufficient for detecting small influenza/ILI waves. Nevertheless, the influenza wave observed at the end of the season was not defined as an epidemic wave because the threshold for detecting an influenza epidemic was not defined previously.

A low or absent influenza/ILI epidemic activity in the 2021–2022 influenza season was also observed in other countries, based on different influenza surveillance systems. In the USA, the weekly percentages of outpatient medical visits due to ILI (fever plus a cough or sore throat) were lower than the basely threshold of 2.5% during the season except in December 2021 (5%) and only 2.6% of the test performed by public health laboratories were positive for influenza, whereas the positivity reached a peak of 26–30% in 2017–2020 [24]. In Denmark, the Influmeter influenza surveillance system including 10,000 participants detected an ILI activity lower than the epidemic threshold during the season [25]. In Canada, the Fluwatchers influenza surveillance system including 10,892 individuals detected weekly percentages of individuals with coughs, which was lower than those detected during prior influenza epidemics [26].

The results obtained in this study could not be affected by changes in health-seeking behavior, based on the comparison of data on prior medical visits, dispensation of medications and prescription of medications in 2021–2022 and prior influenza seasons. Quite similar percentages of ILI patients had received a prior medical visit in the 2021–2022 influenza season (34.8%) and in previous influenza seasons (31–38%) [8,9]. Quite similar dispensation percentages were observed in the 2021–2022 influenza season (93.8%) and in previous influenza seasons (94–98%) [8,9]. Paracetamol and cough medications were dispensed to more than 25% of ILI patients in the 2021–2022 influenza season, while, in the influenza seasons with epidemic activity, four medications (paracetamol, cough medications, ibuprofen and antihistamines) were dispensed to more than 25% of the ILI patients [8]. The dispensation of paracetamol in 2021–2022 (76.6%) was similar to that in the 2017–2020 influenza seasons (72.4–83.8%) [8] and higher than in the 2020–2021 influenza season (71.2%) [9]. The dispensation of oseltamivir in 2021–2022 (0.5%) was similar to that in 2017–2020 (0.1–1%) [8] and higher than that in 2020–2021 (0%) [9]. The dispensation of antibiotics in 2021–2022 (6.6%) was similar to that in 2017–2020 (3.7–7.9%) [8] and higher than that in 2020–2021 (2.8%) [9]. The physicians had prescribed medications to 30.3% of all ILI patients and 38.2% of ILI patients with a negative COVID-19 test in the 2021–2022 influenza season and to 31–38% in previous influenza seasons [8,9].

The results obtained in this study for the ILI epidemic activity, medications dispensed and influenza vaccination effectiveness in the 2021–2022 influenza season could not be affected by the SARS-CoV-2 pandemic for two reasons. First, the analysis carried out for ILI cases with a negative COVID-19 test avoids the potential overestimation of ILI cases due to SARS-CoV-2 infections among ILI cases not undertaking a COVID-19 detection test. Second, the sentinel pharmacies did not report patients with COVID-19 or a positive COVID-19 test and ILI patients who were contacts of patients with COVID-19 or individuals a positive COVID-19 test.

In recent years, several influenza syndromic surveillance systems have been developed [27]. The Triple project-S-AGE assessing nine influenza syndromic surveillance systems developed in Europe found that their success could explain by three factors: (1) Implementation of subnational or regional influenza surveillance systems. (2) Utilization of preclinical data collected before the laboratory confirmation of influenza. (3) Utilization of no clinical information [28]. Another study comparing different influenza syndromic surveillance systems found that their success could be explained by using data from mild and moderate patients and from ILI patients without laboratory confirmation of influenza [29]. Several studies have found that drug sales data analysis could be a useful tool for influenza surveillance [27]. Nevertheless, influenza surveillance systems based on drug sales data analysis are complex and costly and they do not provide precise information about ILI patients, medications dispensed and medications prescribed to ILI patients and influenza vaccination effectiveness.

The involvement of community pharmacies in the prevention and detection of communicable diseases is becoming increasingly important [30,31,32]. Community pharmacies are involved in the prevention and treatment of influenza by dispensing anti-influenza medications and influenza vaccines, reviewing medications and drug interactions, supporting primary health care and specialized services, providing screening tests, providing protective masks and developing health education activities and administering influenza vaccines [1,2,3,4,5,6,30].

Many European countries have introduced changes in legislation to expand the activities related to the prevention and treatment of infectious diseases. In 2020, specially trained pharmacists were involved in administering vaccines in twelve European countries (Belgium, Norway, Germany, Greece, Ireland, Italy, Portugal, Denmark, France, Norway, Switzerland and the United Kingdom) [32,33]. The main reason for implementing vaccination activities in community pharmacies is that they can be important to achieve high percentages of vaccination coverage among adolescents and adults and relieve pressure on the rest of the healthcare system [4,5]. In the United States of America and the United Kingdom, community pharmacies have administered COVID-19 vaccines [34,35].

Influenza surveillance and control activities developed by community pharmacies can be important to achieve high percentages of influenza vaccination coverage and to provide influenza-like illness surveillance data and influenza vaccination data complementary of those provided by traditional influenza surveillance systems. Community pharmacists can increase the influenza vaccination coverage by dispensing and administering influenza vaccines and by developing health education activities [1,2,3,4,5,6]. The pharmacists serving as established advocates and educators as well as qualified providers of vaccinations have a significant role to play in promoting and supporting the influenza vaccination uptake. A recent meta-analysis of randomized controlled trials and observational studies found that vaccination programs with community pharmacies involvement as immunizers and advocators was associated with 14% significantly higher influenza vaccination rates in the United States [4]. The activities developed by the sentinel pharmacies in Spain are important to achieve high percentages of influenza vaccination coverage for several reasons. First, the influenza surveillance data collected by sentinel pharmacies can highlight the importance of influenza vaccination among individuals aged 65 years or more and individuals with chronic conditions. Second, pharmacists can develop health education activities to promote influenza vaccination in the population and among individuals aged 65 years or more and those with chronic conditions. Third, community pharmacies could administer influenza vaccines.

The study has several limitations. First, fifty-three sentinel pharmacies reported ILI cases to the influenza surveillance information system in the 2021–2022 influenza season. More consistent results could have been obtained with a higher number of sentinel pharmacies participating in the study. Nevertheless, 53 sentinel pharmacies could be sufficient to detect the influenza activity, based on the analysis of influenza surveillance data collected in previous influenza seasons [8,9]. Second, the number of ILI cases reported by sentinel pharmacies could be lower than the real number if pharmacies were not able to detect all ILI cases among attended persons. Nevertheless, it should be necessary to develop a complex and costly study to assess the exact number of ILI patients attended by sentinel pharmacies during the influenza season. Third, 52.7% of ILI cases reported by sentinel pharmacies were negative for COVID-19 detection tests. The number of ILI cases with a negative COVID-19 test could be higher if COVID-19 detection tests had been used to detect SARS-CoV-2 infections among all the ILI cases attended by sentinel pharmacies. Nevertheless, the sentinel pharmacies did not have resources for using COVID-19 detection tests in all ILI cases and this limitation was solved by assessing influenza surveillance data collected from sentinel pharmacies for all the ILI cases and for the ILI cases with a negative COVID-19 test.

## 5. Conclusions

The study found a high influenza vaccine effectiveness in preventing ILI among individuals aged 65 years or more and that ILI epidemic activity did not increase significantly during the 2021–2022 influenza season in Catalonia (Spain), based on surveillance data collected by sentinel pharmacies. The sentinel pharmacies provided influenza surveillance data complementary to those provided by traditional influenza surveillance systems.

## Figures and Tables

**Table 1:** Distribution of total influenza-like illness cases (ILI) and influenza-like illness cases with a negative COVID-19 test reported by sentinel pharmacies in Catalonia (Spain) in the 2021–2022 influenza season.

Age	Influenza-Like Illness Cases Reported by Sentinel Pharmacies in the 2021–2022 Influenza Season			
	Total ILI Cases		ILI Cases with a Negative COVID-19 Test	
	No.	% (95% CI)	No.	% (95% CI)
Both sexes				
0–4 years	19	4.7 (2.5–6.9)	9	4.2 (1.3–7.2)
5–14 years	32	8.0 (5.2–10.7)	16	7.5 (3.8–11.3)
15–64 years	315	78.4 (74.2–82.5)	167	78.8 (73.0–84.5)
≥65 years	36	8.9 (4.0–11.9)	20	9.4 (5.3–13.6)
Total	402	100	212	100
Men				
0–4 years	13	9.8 (2.8–10.2)	7	6.8 (1.4–12.1)
5–14 years	18	6.5 (4.8–13.2)	9	8.7 (2.8–14.7)
15–64 years	157	73.9 (72.6–84.4)	81	78.6 (70.2–87.0)
≥65 years	12	9.8 (2.5–9.5)	6	5.8 (0.8–10.8)
Total	200	100	103	100
Women				
0–4 years	6	4.7 (0.4–5.6)	2	1.8 (0.2–6.5)
5–14 years	14	9.4 (3.2–10.7)	7	6.4 (1.4–11.5)
15–64 years	158	69.4 (72.3–84.2)	86	78.9 (70.8–87.0)
≥65 years	24	16.5 (7.2–16.6)	14	12.8 (6.1–19.6)
Total	202	100	109	100

**Table 2:** Influenza vaccination coverage in influenza-like illness (ILI) cases reported by sentinel pharmacies and influenza vaccination effectiveness in individuals aged 65 years or more in Catalonia, Spain, in the 2021–2022 influenza season.

Age	Influenza Vaccination Coverage in ILI Cases Reported by Sentinel Pharmacies and Influenza Vaccination Effectiveness					
	ILI Cases with a Negative COVID-19 test			Total ILI Cases		
	No.	% (95% CI)	*n*	No.	% (95% CI)	*n*
0–4 years	0	0.0 (0.0–33.6)	9	0	0.0 (0.0–17.6)	19
5–14 years	1	6.3 (0.2–30.2)	16	1	3.1 (0.1–16.2)	32
15–64 years	10	6.0 (2.1–9.9)	167	12	3.8 (1.5–6.1)	315
≥65 years	6	30.0 (11.9–54.3)	20	11	30.5 (14.1–47.0)	36
Total	17	8.0 (4.1–11.9)	212	24	6.0 (3.5–8.4)	402
Vaccine Effectiveness (≥65 years)	76.0 (37.5–90.8)			75.4 (49.9–87.9)		

**Table 3:** Percentage of dispensation and percentage of medications to influenza-like illness (ILI) patients with a negative COVID-19 test in Catalonia (Spain) in the 2021–2022 influenza season.

Age	Dispensation of Medications to ILI Cases with a Negative COVID-19 Test			Prescription of Medications to ILI Cases with a Negative COVID-19 Test		
	No.	% (95% CI)	*n*	No.	% (95% CI)	*n*
Both sexes						
0–4 years	8	88.9 (51.8–99.7)	9	12	66.7 (29.9–92.5)	9
5–14 years	14	87.5 (61.6–98.4)	16	6	18.8 (4.0–45.6)	16
15–64 years	156	93.4 (89.3–97.5)	167	92	37.7 (30.1–43.4)	167
≥65 years	19	95.0 (75.1–99.9)	20	12	45.0 (23.1–66.5)	20
Total	197	92.9 (89.2–96.6)	212	122	8.2 (31.4–45.0)	212
Men						
0–4 years	6	85.7 (42.1–99.6)	7	8	57.1 (18.4–90.1)	7
5–14 years	7	77.8 (40.0–97.2)	9	3	22.2 (2.9–60.0)	9
15–64 years	73	90.1 (83.0–97.2)	81	42	35.8 (24.7–46.9)	81
≥65 years	6	100.0 (83.0–97.2)	6	6	66.7 (22.3–95.7)	6
Total	92	89.3 (82.9–95.8)	103	59	37.9 (28.0–47.7)	103
Women						
0–4 years	2	100.0 (15.8–100)	2	4	100.0 (15.8–100)	2
5–14 years	7	100.0 (59.0–100)	7	3	14.3 (0.4–57.9)	7
15–64 years	83	96.5 (90.1–99.3)	86	50	39.5 (28.6–50.4)	86
≥65 years	13	92.9 (66.1–99.5)	14	6	35.7 (12.8–64.9)	14
Total	105	96.3 (90.9–99.0)	109	63	38.5 (28.9–48.1)	109

**Table 4:** Drugs dispensed by sentinel pharmacies and drugs prescribed by physicians to influenza-like illness patients with a negative COVID-19 test in Catalonia (Spain) in the 2021–2022 influenza seasons.

Drug	Drugs Dispensed (*n* = 212)		Drugs Prescribed (*n* = 212)	
	No.	% (95% CI)	No.	% (95% CI)
Paracetamol	162	76.4 (70.5–82.4)	64	30.2 (23.8–36.6)
Ibuprofen	46	21.7 (15.9–27.5)	22	10.4 (6.0–14.7)
Acetylsalicylic acid	1	0.5 (0.0–2.6)	0	0.0 (0.0–1.7)
Cough medication	67	31.6 (25.1–38.1)	19	9.0 (3.8–13.0)
Antihistamines	45	21.2 (14.5–27.0)	16	7.5 (2.7–7.2)
Epinephrine	13	6.1 (2.7–9.6)	2	0.9 (0.1–3.4)
Antibiotic	14	6.6 (3.0–10.2)	14	6.6 (3.0–10.2)
Antiseptic	5	2.4 (0.8–5.4)	1	0.5 (0.0–2.6)
Mucolytic	10	4.7 (1.6–7.8)	8	3.8 (1.0–6.6)
Medicinal plants	5	2.4 (0.8–5.4)	1	0.6 (0.0–2.6)
Bronchodilator	5	2.4 (0.8–5.4)	3	1.4 (0.3–4.1)
Anti-inflammatory	5	2.4 (0.8–5.4)	2	0.9 (0.1–3.4)
Oseltamivir	1	0.5 (0.0–2.6)	1	0.5 (0.0–2.6)

## Data Availability

Data supporting the reported results can be requested from the corresponding author.

## Supplementary Materials

The following supporting information can be downloaded at: https://www.mdpi.com/article/10.3390/vaccines10122140/s1, Table S1: Percentage of influenza-like illness cases reported by sentinel pharmacies that had received a previous medical visit in Catalonia (Spain) in the 2021–2022 influenza season; Table S2: Percentage of dispensation and prescription to all influenza-like illness (ILI) patients in Catalonia (Spain) in the 2021–2022 influenza season; Table S3: Drugs dispensed by sentinel pharmacies and drugs prescribed by physicians to all influenza-like illness patients in Catalonia (Spain) in the 2021–2022 influenza season.

## References

[1] Plans Rubió P. (2022). Role of community pharmacies in the prevention of communicable diseases. Vacunas (Engl. Ed.).

[2] Pharmaceutical Group of European Union (2018). Pharmacy 2030: A Vision for Community Pharmacy in Europe.

[3] Hansen R.N., Nørgaard L.S., Hedegaard U., Søndergaard L., Servilieri K., Bendixen S., Rossing C. (2021). Integration of and Visions for Community Pharmacy in Primary Health Care in Denmark. Pharm. Pract. (Granada).

[4] Le L.M., Veettil S.K., Donaldson D., Kategeaw W., Hutubessy R., Lambach P., Chaiyakunapruk N. (2022). The impact of pharmacist involvement on immunization uptake and other outcomes: An updated systematic review and meta-analysis. J. Am. Pharm. Assoc..

[5] Czech M., Balcerzak M., Antczak A., Byliniak M., Piotrowska-Rutkowska E., Drozd M., Juszczyk G., Religioni U., Vaillancourt R., Merks P. (2020). Flu Vaccinations in Pharmacies—A Review of Pharmacists Fighting Pandemics and Infectious Diseases. Int. J. Environ. Res. Public Health.

[6] Nusair M.B., Arabyat R., Mukattash T.L., Alhamad H., Abu Ghaida M.T., Momani M.Y. (2020). Pharmacists’ Perspectives on Providing the Influenza Vaccine in Community Pharmacies: A Qualitative Study. Risk Manag. Healthc. Policy.

[7] Ministerio de Sanidad (2019). Guía de Procedimientos Para la Vigilancia de la Gripe en España.

[8] Plans-Rubió P., Jambrina A.M., Carmona G., Rabanal M., Jané M., Rius P. (2021). Influenza syndromic surveillance based on sentinel pharmacies in Catalonia (Spain) in 2017–2020. Int. J. Mod. Pharm. Res..

[9] Plans-Rubió P., Jambrina A.M., Rius P., Carmona G., Rabanal M., Girones M. (2022). Absence of the influenza epidemic in the 2020-2021 influenza seaon in Catalonia, Spain, based on surveillance data collected by sentinel pharmacies. Int. J. Mod. Pharm. Res..

[10] Jambrina A.M., Rams N., Rius P., Perelló M., Gironès M., Pareja C., Pérez-Cano F.J., Franch À., Rabanal M. (2022). Creation and Implementation of a New Sentinel Surveillance Model in Pharmacy Offices in Southern Europe. Int. J. Environ. Res. Public Health.

[11] Koppeschaar C.E., Colizza V., Guerrisi C., Turbelin C., Duggan J., Edmunds W.J., Kjelsø C., Mexia R., Moreno Y., Meloni S. (2017). Influenzanet: Citizens Among 10 Countries Collaborating to Monitor Influenza in Europe. JMIR Public Health Surveill..

[12] Vega T., Lozano J.E., Merhoff T., Snacken R., Mott J., Ortiz de Lejarazu R., Nunes B. (2013). Influenza surveillance in Europe: Establishing epidemic thresholds by the moving epidemic method. Influenza Other Respir. Viruses.

[13] Asociación Española de Pediatría (2022). Coberturas de Vacunación en la Temporada 2021–2022.

[14] Farrington C.P. (1993). Estimation of vaccine effectiveness using the screening method. Int. J. Epidemiol..

[15] Institut d’Estadística de Catalunya (IDESCAT) (2022). Anuari Estadístic de Catalunya.

[16] Uchida M., Kaneko M., Hidaka Y., Yamamoto H., Honda T., Takeuchi S., Saito M., Kawa S. (2016). Effectiveness of vaccination and wearing masks on seasonal influenza in Matsumoto City, Japan, in the 2014/2015 season: An observational study among all elementary schoolchildren. Prev. Med. Rep..

[17] Huang Q.S., Wood T., Jelley L., Jennings T., Jefferies S., Daniells K., Nesdale A., Dowell T., Turner N., Campbell-Stokes P. (2021). Impact of the COVID-19 nonpharmaceutical interventions on influenza and other respiratory viral infections in New Zealand. Nat. Commun..

[18] Centers for Disease Control and Prevention (CDC) Interim US Flu Vaccine Effectiveness (VE) Data for 2021–2022. Reviewed 3 August 2022. https://www.cdc.gov/flu/vaccines-work/2021-2022.html.

[19] Goad J., Influenza Activity and Vaccine Effectiveness during the 2021–2022 Influenza Season Pharm. Times 2022..

[20] World Health Organization (WHO) (2017). Evaluation of Influenza Vaccine Effectiveness: A Guide to the Design and Interpretation of Observational Studies.

[21] Gilca R., Amini R., Douville-Fradet M., Charest H., Dubuque J., Boulianne N., Skowronski D.M., De Serres G. (2014). Other respiratory viruses are important contributors to adult respiratory hospitalizations and mortality even during peak weeks of the influenza season. Open Forum. Infect. Dis..

[22] Sub-Direcció General de Vigilància Epidemiològica (2022). Pla D’informació de les Infeccions Respiratòries Agudes A Catalunya. Temporada 2021–2022.

[23] Sub-Direcció General de Vigilància Epidemiològica (2022). Pla D’informació de les Infeccions Respiratòries Agudes A Catalunya 2021–2022.

[24] Centers for Disease Control and Prevention (CDC) (2022). Weekly U.S. Influenza Surveillance Report.

[25] Government of Canada FluWatch Report: May 15 to May 21, 2022 (week 20). https://health/services/publications/diseases-conditions/fluwatch/2021-2022/week-20-may-15-may-21-2022.html.

[26] Statems Serum Institut Influenza Season 2021/2022. https://en.ssi.dk/surveillance-and-preparedness/surveillance-in-denmark/annual-reports-on-disease-incidence/influenza-season-2021-2022.

[27] Pivette M., Mueller J.E., Crépey P., Bar-Hen A. (2014). Drug sales data analysis for outbreak detection of infectious diseases: A systematic literature review. BMC Infect. Dis..

[28] Ziemann A., Fouillet A., Brand H., Krafft T. (2016). Success Factors of European Syndromic Surveillance Systems: A Worked Example of Applying Qualitative Comparative Analysis. PLoS ONE.

[29] Dailey L., Watkins R.E., Plant A.J. (2007). Timeliness of data sources used for influenza surveillance. JAMIA.

[30] Pharmaceutical Group of the European Union (PGEU) Position Paper on the Role of Community Pharmacists in COVID-19—Lessons Learned from the Pandemic. https://www.pgeu.eu/wp-content/uploads/2020/03/PGEU-Position-Paper-on-on-the-Lessons-Learned-from-COVID-19-ONLINE.pdf.

[31] OECD (2020). Beyond Containment: Health Systems Responses to COVID-19 in the OECD. OECD. https://read.oecd-ilibrary.org/view/?ref=119_119689-ud5comtf84&title=Beyond_Containment:Health_systems_responses_to_COVID-19_in_the_OECD.

[32] WHO (2020). Strengthening the Health System Response to COVID-19.

[33] Verjee Z., Duggan C., Swaminathan S., Gellin B., Piervincenzi R., Etienne C. Quality, Speed & Equity: Delivering COVID-19 Vaccines to the World. Virtual Webinar; 15 March 2021. https://zoom.us/webinar/register/WN_S69BcKzhRDy8ll1I1SRuxw.

[34] Royal Pharmaceutical Society (2020). RPS Statement on the Role of Pharmacists in the COVID-19 Vaccination Programme.

[35] CDC Pharmacies Participating in COVID-19 Vaccination. https://www.cdc.gov/vaccines/covid-19/retail-pharmacy-program/participating-pharmacies.html.

