# Supplementary material for: High Influenza Vaccine Effectiveness and Absence of Increased Influenza-like-Illness Epidemic Activity in the 2021–2022 Influenza Season in Catalonia (Spain) Based on Surveillance Data Collected by Sentinel Pharmacies
Source: Vaccines (Basel). 2022 Dec 14;10(12):2140. doi: 10.3390/vaccines10122140 (PMC9783856; doi:10.3390/vaccines10122140)
Supplement: Supplementary file 1 [file vaccines-10-02140-s001.zip › vaccines-2054744-supplementary.pdf]

Supplement Materials

Table S1. Percentage of influenza-like illness cases reported by sentinel pharmacies that had received a previous medical visit in Catalonia (Spain) in the 2021-2022 influenza season

Table S2. Percentage of patients with influenza-like illness cases illness (ILI) that sentinel pharmacies dispensed medications and physicians prescribed medications in Catalonia (Spain) in the 2021-2022 influenza season

Table S3. Drugs dispensed by sentinel pharmacies and drugs prescribed by physicians to influenza-like illness patients in Catalonia (Spain) in the 2021−2022 influenza season

Table S1. Percentage of influenza-like illness cases reported by sentinel pharmacies that had received a previous medical visit in Catalonia (Spain) in the 2021-2022 influenza

Age	Influenza-like illness (ILI) cases reported by sentinel pharmacies in the 2021−2022 influenza season					
	Total ILI cases			ILI cases with a negative COVID-19 test result		
	No.	% (95% CI)	n	No.	% (95% CI)	n
Both sexes						
0-4 years	13	68.4 (43.4−87.4)	19	6	66.7 (29.9−92.5)	9
5-14 years	7	21.9 (6.0−37.8)	32	4	25.0 (7.3−52.4)	16
15-64 years	107	34.0 (28.6−39.4)	315	74	44.3 (36.5−52.1)	167
≥ 65 years	13	36.1 (19.5−53.2)	36	10	50.0 (25.6−74.4)	20
Total	140	34.8 (30.0−39.6)	402	94	44.3 (37.4−51.3)	212
Men						
0-4 years	9	69.2 (38.6−90.9)	13	7	57.1 (18.4−90.1)	7
5-14 years	3	16.7 (3.6−41.4)	18	9	22.2 (22.2−60.0)	9
15-64 years	47	29.9 (22.4−37.4)	157	81	40.7 (29.4−52.1)	81
≥ 65 years	6	50.0 (21.1−78.9)	12	6	66.7 (22.8−95.7)	6
Total	65	32.5 (25.8−39.2)	200	103	41.7 (31.7−51.8)	103
Women						
0-4 years	4	66.7 (22.3−95.7)	6	2	100.0 (15.8−100)	2
5-14 years	4	28.6 (8.4−58.1)	14	7	28.6 (3.7−71.0)	7
15-64 years	60	38.0 (30.1−45.6)	158	86	47.7 (36.5−58.8)	86
≥ 65 years	7	29.2 (12.1−51.1)	24	14	42.9 (17.7−71.1)	14
Total	75	37.1 (30.2−44.0)	202	109	46.8 (37.0−56.6)	109

Table S2. Percentage of patients with influenza-like illness cases illness (ILI) that sentinel pharmacies dispensed medications and physicians prescribed medications in Catalonia (Spain) in the 2021-2022 influenza season

	ILI cases with dispended medications			ILI cases with prescribed medications		
Age	No.	% (95% CI)	n	No.	% (95% CI)	n
Both sexes						
0-4 years	17	89.5 (66.9−98.7)	19	12	63.2 (38.4−83.7)	19
5-14 years	30	93.8 (79.2−99.2)	32	6	18.8 (7.2−36.4)	32
15-64 years	296	94.0 (91.2−96.8)	315	92	29.2 (24.0−34.4)	315
≥ 65 years	34	94.4 (81.3−99.3)	36	12	33.3 (16.5−50.1)	36
Total	377	93.8 (91.3−96.3)	402	122	30.3 (25.7−35.0)	402
Men						
0-4 years	12	92.3 (64.0−99.8)	13	8	61.5 (31.6−86.1)	13
5-14 years	16	88.9 (65.3−98.6)	18	3	16.7 (3.6−41.4)	18
15-64 years	146	93.0 (88.7−97.3)	157	42	26.7 (19.5−34.0)	157
≥ 65 years	12	100.0 (73.5−100)	12	6	50.0 (21.1−78.9)	12
Total	186	93.0 (89.2−86.8)	200	59	29.5 (22.9−36.1)	200
Women						
0-4 years	5	83.3 (35.9−99.0)	6	4	66.7 (22.8−95.7)	6
5-14 years	14	100.0 (76.8−100)	14	3	21.4 (4.7−50.8)	14
15-64 years	150	94.9 (91.2−98.7)	158	50	31.6 (24.1−39.2)	158
≥ 65 years	22	91.7 (73.1−99.0)	24	6	25.0 (9.8−46.7)	24
Total	191	94.6 (91.2−97.9)	202	63	31.2 (24.5−37.6)	202

Table S3. Drugs dispended by sentinel pharmacies and drugs prescribed by physicians to influenza-like illness patients in Catalonia (Spain) in the 2021−2022 influenza season

Drug	Drugs dispensed (n = 402)		Drugs prescribed (n = 402)	
	No.	% (95% CI)	No.	% (95% CI)
Paracetamol	308	76.6 (72.3−80.9)	96	23.9 (19.6−28.2)
Ibuprofen	83	20.6 (16.6−24.7)	37	9.2 (6.2−12.1)
Acetylsalicylic acid	7	1.7 (0.3−3.1)	1	0.2 (0.0−1.4)
Cough medication	110	27.4 (22.9−31.8)	31	7.7 (5.0−10.4)
Antihistamines	89	22.1 (18.0−26.3)	20	5.0 (2.7−7.2)
Epinephrine	32	8.0 (5.2−10.7)	3	0.7 (0.1−2.2)
Antibiotic	19	4.7 (2.5−6.9)	19	4.7 (2.5−6.9)
Antiseptic	11	2.7 (1.0−4.4)	2	0.5 (0.0−1.8)
Mucolytic	27	6.7 (4.1−9.3)	11	2.7 (1.0−4.4)
Medicinal plants	11	2.7 (1.0−4.4)	1	0.2 (0.0−1.4)
Bronchodilator	8	2.0 (0.5−3.5)	4	1.0 (0.3−2.5)
Anti-inflammatory	7	1.7 (0.3−3.1)	3	0.7 (0.1−2.2)
Oseltamivir	1	0.2 (0.0−1.4)	1	0.2 (0.0−1.4)
